# Health care utilization in general practice after HPV vaccination—A Danish nationwide register-based cohort study

**DOI:** 10.1371/journal.pone.0184658

**Published:** 2017-09-08

**Authors:** Lene Wulff Krogsgaard, Claus Høstrup Vestergaard, Oleguer Plana-Ripoll, Tina Hovgaard Lützen, Mogens Vestergaard, Morten Fenger-Grøn, Bodil Hammer Bech, Dorte Rytter

**Affiliations:** 1 Research Unit for General Practice, Department of Public Health, Aarhus University, Aarhus, Denmark; 2 Section for Epidemiology, Department of Public Health, Aarhus University, Aarhus, Denmark; 3 National Centre for Register-based Research, Aarhus BSS, Aarhus University, Aarhus, Denmark; 4 Section for General Medical Practice, Department of Public Health, Aarhus University, Aarhus, Denmark; Universidade Estadual de Maringa, BRAZIL

## Abstract

**Objective:**

The Human Papillomavirus (HPV) vaccine has increasingly been suspected of adverse effects in Denmark since 2013. By using consultations with the general practitioner (GP) as an indicator for morbidity, this study aims to examine the association between HPV vaccination and morbidity in girls in the Danish childhood immunization program.

**Methods:**

The study is a nationwide register-based cohort study. Both the HPV and the Measles, Mumps and Rubella (MMR) vaccines were offered to 12-year-old girls in Denmark in the study period (2008–2015). Therefore, both vaccines were included as exposures to allow differentiation between potential effects. This resulted in four exposure groups: HPV only vaccinated, HPV+MMR vaccinated, MMR only vaccinated, and Non-vaccinated girls. Outcomes were: daytime consultation rates and frequent GP attendance (> 7 annual GP consultations). We estimated consultation rates by negative binomial regressions analysis and frequent GP attendance by logistic regression analysis. Both analyses were stratified on the years 2008–2013 versus 2014.

**Results:**

The study included 214,240 girls born in 1996–2002. All vaccinated groups consulted the GP more often than the non-vaccinated group, both before and after the vaccination. After the vaccination, an increase in consultations was observed for all three vaccinated groups; most distinct for girls vaccinated in 2014. For girls vaccinated before 2014, we found a slightly higher risk of frequent GP attendance after vaccination in the HPV only group compared to the non-vaccinated group, whereas in 2014, frequent GP attendance was seen for all three vaccinated groups; most substantial for the MMR only vaccinated group.

**Conclusion:**

In this study, no exclusive increase in health care utilization was detected after HPV vaccination. However, a general difference in the health care utilization pattern was found between vaccinated and non-vaccinated girls, which increased after the time of vaccination, primarily for girls vaccinated after 2013.

## Introduction

Cervix cancer is the second most common cancer in women worldwide, accounting for 270,000 deaths in 2012 [1,2]. Human papillomavirus (HPV) is the cause of virtually all cervical cancer cases [3,4], and more than sixty out of 195 countries worldwide have included the HPV vaccine in their national immunization programs [5]. The HPV vaccination was introduced in the Danish national childhood immunization program in 2009. Since then, the vaccination has been offered to all 12-year-old girls [6]

Large international randomized controlled trials showed that the HPV vaccine was safe and well-tolerated [7,8] and subsequent epidemiological studies have not found a higher risk of autoimmune or neurological diseases in vaccinated girls or women compared to un-vaccinated [9–11]. However, the HPV vaccination program has been challenged in Denmark since 2013 due to an increasing number of suspected adverse effects of the HPV vaccine followed by an intense media attention and public debate [12]. Some of the reported symptoms have been classified as postural orthostatic tachycardia syndrome (POTS), chronic fatigue syndrome, long-lasting dizziness, headache, syncope, seizures, abdominal pain, joint and muscle pain, and cognitive dysfunction [13,14].

The Global Advisory Committee on Vaccine Safety, which was set up by the World Health Organization (WHO), reported in 2015 that no safety issues that would alter the recommendation of the HPV vaccination had been found [15]. As the reported adverse effects are very heterogeneous, and most of them do not have a specific hospital diagnosis code, this is a complex area, which is difficult to study. It has, therefore, been discussed whether the diagnosis-based epidemiological studies fully cover these possible adverse effects.

In Denmark, all citizens have free and direct access to the general practitioner (GP). The GP is the first point of contact in the health care system, and many health problems are handled and treated by the GP, who acts as gatekeeper to secondary care, e.g. through referral to specialists and hospitals [16,17]. Girls experiencing possible adverse effects of the HPV vaccine are most likely to first seek help at their GP and we expect that healthcare utilization at the GP will reflect potential health effects due to the HPV vaccine. The aim of the current study was, therefore, to examine the association between the HPV vaccine and primary health care utilization as indicator for increased morbidity among girls included in the Danish childhood immunization program. As the Measles, Mumps and Rubella (MMR) vaccination was offered to girls at the same age as the HPV vaccination, information on MMR vaccination was included in the study.

## Materials and methods

### Study population

The study was designed as a register-based cohort study. Every citizen in Denmark is registered with a unique 10-digit civil registration number (CRN) [18]. This number was used to identify the study population and link information at the individual level between the registers used in the study. In the Danish National Patient Register [19], we identified all girls born in Denmark between 1996 and 2002 (n = 226,146). From the Danish Civil Registration System [18], we obtained information on emigration and death, and girls who were still alive and living in Denmark at their 14^th^ birthday or at the end of follow-up on 31 December 2015, whichever came first, were included in the study (n = 214,424). Girls with missing information on region of residence were excluded (n = 184), and the final study population then consisted of 214,240 girls.

### Information on exposure and outcome

Information on both exposure and outcome was obtained from the Danish National Health Insurance Service Register (NHSR) [20]. All primary health care services provided to citizens in Denmark are registered in the NHSR with specific codes. The registrations are based on a fee-for-service payment of the GP, and registered records are virtually complete. This makes it possible to follow the individual contacts with primary care over time. The register includes information on the year and week in which the service is provided [20].

### Exposure

The Danish immunization program includes vaccines against nine different infectious diseases given at different ages through childhood. In addition, girls are offered the HPV vaccine. The vaccinations are provided by the GP and is free of charge [21]. The HPV and MMR vaccinations were both offered to 12-year-old girls as part of the Danish immunization program in the period 2009–2016. The main exposure in the study was the HPV vaccine, but the MMR vaccine was also considered as an exposure in order to examine potential effect modification.

#### HPV vaccine

In January 2009, the HPV vaccination was introduced in the national childhood immunization program and offered to all 12-year-old girls [6]. In the period between January 2009 and August 2014, girls were vaccinated with the quadrivalent Gardasil vaccine three times within a year. After this, the vaccination program was changed, and Gardasil was only administered twice. The vaccines were injected by the GP and registered in the NHSR (service codes: 8328, 8329, 8330 or 8334, 8335, 8336). In the current study, a girl was categorized as exposed to HPV vaccine if one of the HPV vaccine service codes appeared in the register before her 14^th^ birthday or before the end of follow-up, whichever came first.

#### MMR vaccine

From 1987 until April 2016, the MMR vaccine was offered to children in Denmark as part of the national childhood immunization program at fifteen months and twelve years of age. After the inclusion of the HPV vaccine in the national childhood immunization program in 2009, the MMR and HPV vaccine were both offered to 12-year-old girls at the same time. In this study, a girl was categorized as MMR vaccinated if either the MMR vaccine service code provided for 12-year-old children was registered (8612) or the MMR vaccine service code for the vaccination given at fifteen months was registered after the age of eleven years (8601).

#### Exposure status

The exposure was categorized into four mutually exclusive groups: HPV only vaccinated, HPV+MMR vaccinated, MMR only vaccinated, and Non-vaccinated (neither HPV- nor MMR vaccinated). The date of vaccination was defined as a random date in the week of the first HPV vaccination according to the NHSR for the two HPV vaccinated groups. For the MMR only vaccinated girls, it was defined as a random date in the week of the MMR vaccination. The non-vaccinated girls do not have a date of vaccination, and this group served as a reference group representing heath care utilization at a given age and for a given calendar period.

### Outcome

The outcome in the study was primary health care utilization measured as face-to-face daytime consultation rates (hereafter referred to as *consultations*) and high frequency of GP attendance (hereafter referred to as *frequent GP attendance*). Frequent GP attendance was defined as more than 7 daytime face-to-face consultations during the year following the vaccination/index date. Information about consultations at the GP (service code for consultations: 0101) two years before and two years after the date of vaccination was obtained from the NHSR. A vaccination is not supposed to be registered in addition to a consultation code, unless an actual consultation at the GP has taken place. A high number of girls had consultation codes (0101) registered in the same week as a vaccination. Therefore, such consultation was disregarded as it was considered as either a registration error or a minor health concern that did not prevent the GP from vaccinating the girl.

### Covariates

Potential confounders were all selected a priori. Information on age, region of residence, ethnicity, birth order, type of household, parental education and socioeconomic status was obtained from Statistics Denmark [22]. Information on parental covariates was obtained for the year before the date of vaccination for the vaccinated girls and the year before the 12^th^ birthday for the non-vaccinated girls. However, when data was missing, data from two years before the date of vaccination/12^th^ birthday was used.

### Statistics

Negative binomial regression models were used to estimate incidence rate ratios (IRRs) and 95% confidence intervals (CIs) comparing the rate of face-to-face consultations per three months for each of the three vaccinated groups compared to the consultation rate for a group of non-vaccinated girls. This was calculated for the time period from two years before until two years after time of vaccination. We used categorical covariates as presented in Table 1 to ensure that estimates were adjusted for age, calendar year, ethnicity, month of vaccination, region of residence, birth order, type of household, parental education and socioeconomic status. As the HPV vaccination coverage decreased steeply in Denmark in 2014 and 2015 [18,19], a sub-analysis stratifying on year of first vaccination (2008–2013 versus 2014–2015) was performed.

**Table 1:** Demographic characteristics of the study population according to vaccination status.

Vaccination status	None*	HPV+MMR	HPV	MMR	Total
**n (%)**					
Overall (2007–2015)	11,755 (5.5)	175,425 (81.9)	15,938 (7.4)	11,122 (5.2)	214,240
2007–2013	8,090 (4.5)	150,205 (84.4)	12,099 (6.8)	7585(4.3)	177,979
2014–2015	3,665 (10.1)	25,220 (69.5)	3839 (10.6)	3537 (9.8)	36,261
**Age at vaccination**					
11	-	22,877 (13.0)	1,316 (8.3)	2,304 (20.7)	-
12	11,755(100)*	138,603 (79.0)	12,693 (79.6)	7808 (70.2)	-
13	-	13,937 (7.9)	1,928 (12.1)	1,010 (9.1)	-
14	-	10 (0.1)	< 5 (0.0)	0 (0.0)	-
**Year of vaccination**					
2007–2009	-	42,169 (24.0)	2,990 (18.8)	3,597 (32.3)	-
2010–2011	-	58,049 (33.1)	3,954 (24.8)	2,105 (16.9)	-
2012–2013	-	49,840 (28.4)	5,137 (32.3)	3,544 (18.9)	-
2014–2015	-	25,367 (14.5)	3,857 (24.1)	3,544 (31.9)	-
**Season of vaccination**					
Spring	-	50,232 (28.6)	4,270 (26.8)	3,415 (30.7)	-
Summer	-	37,330 (21.3)	3,609 (22.6)	2,517 (22.6)	-
Autumn	-	43,017 (24.5)	3,985 (25.0)	3,116 (28.0)	-
Winter	-	44,846 (25.6)	4,074 (25.6)	2,074 (18.7)	-
**Birth order**					
1	4,577 (38.9)	74,977 (42.7)	6,920 (43.4)	4,690 (42.2)	91,064
2	4,170 (35.5)	67,085 (38.3)	5,960 (37.4)	3,935 (35.4)	81,159
3	1960 (16.7)	25,342 (14.4)	2,243 (14.1)	1,741 (15.7)	31,286
+4	1,048 (8.9)	8,121 (4.6)	815 (5.1)	756 (6.7)	10,740
**Ethnicity of the girl**					
Danish	10,899 (92.7)	161,738 (92.2)	14,449 (90.7)	9,738 (87.6)	196,824
Descendant of immigrants	856 (7.3)	13,687 (7.8)	1,489 (9.3)	1,384 (12.4)	17,416
**Region of residence**					
Northern Denmark	920 (7.8)	19,050 (10.9)	1,247 (7.8)	1,150 (10.3)	22,367
Central Denmark	2,045 (17.4)	42,658 (24.3)	3,999 (25.1)	2,279 (20.5)	50,981
Southern Denmark	3,129 (26.6)	39,491 (22.5)	2,930 (18.4)	2,711 (24.4)	48,261
Capital	3,505 (29.4)	47,528 (27.1)	5,255 (33.0)	3,162 (28.4)	59,450
Zealand	2,156 (18.3)	26,698 (15.2)	2,507 (15.7)	1,820 (16.4)	33,181
**Type of household**					
Father only	24 (0.2)	269 (0.2)	26 (0.2)	13 (0.1)	332
Mother only	3,009 (25.6)	31,816 (18.1)	3,150 (19.8)	2,444 (22.0)	40,419
Married parents	5,945 (50.6)	111,938 (63.8)	9,682 (60.7)	6,468 (58.2)	134,033
Other couples	1,843 (15.7)	22,173 (12.6)	2,062 (12.9)	1,458 (13.1)	27,536
Several families in household	934 (7.9)	9,229 (5.3)	1,018 (6.4)	739 (6.6)	11,920
**Socioeconomic position of family**					
Business owner/co-working spouse	932 (7.9)	11,580 (6.6)	1,112 (7.0)	814 (7.3)	14,438
Top manager/employee with high degree	2,349 (20.0)	40,373 (23.0)	3,833 (24.0)	2,313 (20.8)	48,868
Employee with middle degree	1,743 (14.8)	34,264 (19.5)	2,825 (17.7)	1,829 (16.4)	40,661
Employee with basic degree	2,912 (24.8)	48,178 (27.5)	4,089 (25.7)	2,854 (25.7)	58,033
Remaining employees	1,757 (14.9)	24,286 (13.8)	2,148 (13.5)	1,585 (14.3)	29,776
Unemployed	1,348 (11.5)	10,545 (6.0)	1,279 (8.0)	1,105 (9.9)	14,277
Pensioners	445 (3.8)	4,325 (2.5)	456 (2.9)	403 (3.6)	5,629
Students	106 (0.9)	497 (0.3)	63 (0.4)	65 (0.6)	731
Other	168 (1.4)	1,350 (0.8)	131 (0.8)	148 (1.3)	1,792
Unknown	0 (0.0)	27 (0.0)	<10 (0.0)	<10 (0.1)	<40
**Highest educated parent**					
Primary	29 (0.2)	454 (0.3)	48 (0.3)	53 (0.5)	569
Lower secondary	1,624 (13.8)	13,734 (7.8)	1,453 (9.1)	1,229 (10.5)	17,992
Upper secondary	4,928 (41.9)	76,883 (43.8)	6,717 (42.1)	4,854 (41.3)	93,207
Short cycle tertiary	670 (5.7)	12,605 (7.2)	994 (6.2)	782 (6.7)	15,016
Bachelor or equivalent	2,749 (23.4)	45,963 (26.2)	4,090 (25.8)	2,862 (24.3)	55,553
Master or equivalent	1,496 (12.8)	22,186 (12.6)	2,266 (14.2)	1,561 (13.3)	27,427
Doctoral or equivalent	185 (1.6)	2,622 (1.5)	261 (1.6)	166 (1.4)	3,226
Not elsewhere classified	49 (0.4)	692 (0.4)	77 (0.5)	89 (0.8)	870
Unknown	25 (0.2)	286 (0.2)	32 (0.2)	156 (1.2)	380

*The covariates for the non-vaccinated group of girls are estimated at the 12th birthday

All P-values for difference between groups are < 0.0001

In a supplementary analysis, we studied the association between HPV vaccination and frequent GP attendance. The vaccinated girls were matched on birthdate with non-vaccinated girls. The non-vaccinated girls were allocated an index date equal to the vaccination date of the matched vaccinated girl. The odds ratio (OR) of frequent GP attendance among vaccinated girls was calculated using a logistic regression analysis, with adjustment for all combinations of calendar time of vaccination and age at vaccination, as presented in Table 1. The estimates were also adjusted for prior health care attendance (continuously), ethnicity, region of residence, birth order, type of household, parental education and socioeconomic status. The analysis of frequent GP attendance was stratified on years (2008–2013 versus 2014). Girls who had the HPV vaccination in 2015 were excluded from this analysis as it required one year of follow-up. The adjusted results were presented with 95% CIs.

In all performed analyses, a cluster-robust variance estimation was applied to account for dependence between repeated observations on the same subjects. The statistical analyses were performed in Stata 13.1 (Stata Corporation, College Station, Texas). A two-sided p-value of 0.05 or less was considered statistically significant.

### Ethics

This study was approved by the Danish Data Protection Agency (j.no. 2015-57-0002). According to the Committee on Health Research Ethics in the Central Denmark Region, no ethical approval was required for this study.

## Results

### Characteristics of participants

The characteristics of the 214,240 participants are shown in Table 1. From 2008 through 2013, a total number of 177,979 girls were included of whom 150,205 (84.4%) were HPV+MMR vaccinated, 12,099 (6.8%) were HPV only vaccinated and 7,585 (4.3%) were MMR only vaccinated. Hence 8,090 (4.5%) of the girls were not registered with any of the vaccinations and were considered non-vaccinated. From 2014 through 2015, there was a total number of 36,261 participants. In this period, the numbers were 25,220 (69.5%) for HPV+MMR, 3,839 (10.6%) for HPV only, and 3,537 (9.8%) for MMR only. Hence 3,665 (10.1%) were non-vaccinated. Both before and after 2013, vaccinated girls were more likely than non-vaccinated girls to have parents who were married and who had higher socioeconomic position. Non-vaccinated girls had the lowest mean number of consultations during the entire period. In our study population, the mean age for HPV and MMR vaccination was 12.37 and 12.16, respectively. Of the girls receiving both vaccines 31% were vaccinated at different occasions and in most of these cases (85%) the girls had received the MMR vaccine before the HPV vaccine (median 225 days). Girls receiving the MMR only vaccine were vaccinated on average 40 days earlier than girls receiving the HPV only vaccine.

### Consultations at the General Practitioner

**Fig 1:**
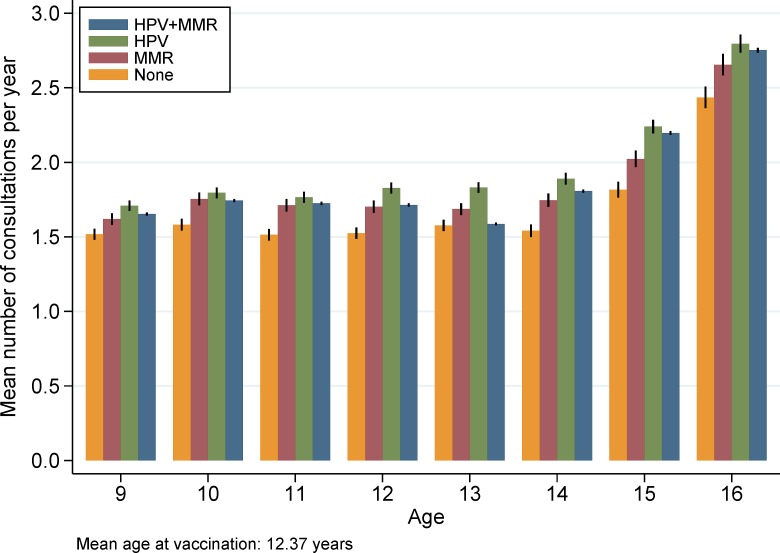
Mean number and 95% CIs of daytime GP face-to-face consultations by age according to vaccination status.

The mean number of consultations at the GP is visualized by age and vaccination status in Fig 1. The mean number of visits at the GP varied from 1.5 visits a year for nine-year-old non-vaccinated girls to 2.8 times a year for the sixteen-year-old HPV only vaccinated girls. All groups had stable consultation rates until age fourteen and progressively increasing (although varying) consultation rates after the age of fourteen. The mean number of consultations was generally highest in the group of HPV only vaccinated girls followed by the HPV+MMR group and the MMR only group.

**Fig 2:**
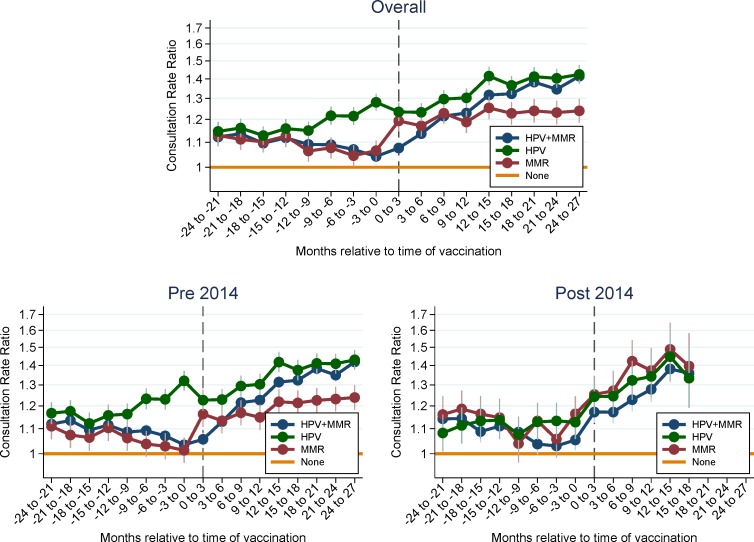
GP consultation rate ratios and 95% CIs (per three months) by vaccination status, two years before/after the time of vaccination.

In the adjusted analysis, higher consultation rates were observed for all three groups of vaccinated girls compared to the group of non-vaccinated girls, both before and after the time of vaccination (Fig 2). There was a tendency to a decrease in consultation rate ratios in the nine months before vaccination for the HPV+MMR and MMR only vaccinated girls, whereas an increase was observed for the HPV only vaccinated group, relative to the non-vaccinated group of girls. After the time of vaccination an increase in consultation rate ratios was observed for the three groups of vaccinated girls; this was most distinct for the HPV only and the HPV+MMR vaccinated groups. In the stratified analysis, the consultation rate ratios for those vaccinated within the period from 2008 through 2013 were very similar to the overall analysis. In 2014 the three vaccinated groups had a similar consultation pattern both before and after time of vaccination with a tendency towards a steeper increase in consultation rate ratios after vaccination, particularly for the MMR group.

### Frequent GP attendance

Table 2 presents the results of the analysis concerning frequent GP attendance stratified on year of vaccination. The percentage of frequent attenders among the included girls was approximately 2–3%. The percentage of frequent attenders was very similar for the HPV only and HPV+MMR vaccinated groups in the two periods, whereas the percentage of frequent attenders decreased for the non-vaccinated girls and increased for the MMR only vaccinated girls in 2014. For those vaccinated from 2008 through 2013, we found a slightly higher risk of frequent GP attendance after vaccination in the HPV only group compared to the non-vaccinated group, which was, however not statistically significant. In contrast, for those vaccinated in 2014, an indication of higher OR of frequent GP attendance was detected for all three vaccination groups; this was especially seen in the group of MMR only vaccinated girls who had an OR of 1.41 (CI: 1.01–1.99).

**Table 2:** ORs and 95% CI of frequent (>7 times) GP attendance during daytime in the 12 months after HPV vaccination according to vaccination status for girls born 1996–2002 in Denmark stratified by period.

Year	2008–2013		2014	
Vaccination status	Percentage of frequent GP users	OR (95% CI) Adjusted*	Percentage of frequent GP users	OR (95% CI) Adjusted*
Non-vaccinated	2.21	1.00 (reference)	1.96	1.00 (reference)
HPV only vaccinated	2.61	1.16 (0.97–1.39)	2.56	1.26 (0.90–1.76)
HPV+MMR vaccinated	2.30	1.01 (0.87–1.17)	2.33	1.23 (0.97–1.58)
MMR only vaccinated	2.19	0.89 (0.72–1.10)	2.82	1.41 (1.01–1.99)

*Adjusted for health care attendance before vaccination, calendar time, age, ethnicity, birth order, region of residence, parental type of household, parental education, and socioeconomic position.

## Discussion

This nationwide population-based cohort study investigated the primary health care utilization as an indicator for increased morbidity after HPV vaccination among girls included in the Danish national immunization program. Overall, the study found that vaccinated girls in all groups had higher consultation rates than non-vaccinated girls both before and after vaccination. The consultation rate ratios, however, tended to increase after vaccination. This was evident in all three vaccination groups, but most distinct for the HPV only and the HPV+MMR vaccinated groups. The consultation rate ratios for those vaccinated in the period from 2008 through 2013 were very similar to the overall analysis, whereas the increase in consultation rate ratios after vaccination tended to be steeper for those vaccinated after 2014. In addition, we observed a higher probability of frequent GP attendance in the year following vaccination for all girls vaccinated in 2014 compared to non-vaccinated girls. This increased probability of frequent attendance in the vaccinated groups in 2014 was possibly partly due to a decrease in the percentage of frequent attenders in the non-vaccinated group.

In the study, no exclusive association between the HPV vaccine and increased health care utilization following vaccination was detected, but a general difference in the health care utilization was found between vaccinated and non-vaccinated 12-year-old girls. Although the results cannot exclude that vaccination is associated with increased morbidity, the similar results for all vaccinated groups do not indicate any specific concerns about the HPV vaccine. Both the steeper increase in consultation rate ratios observed for those vaccinated in 2014–15 and the higher ORs for frequent attendance observed for all three groups vaccinated in 2014, but not for those vaccinated earlier, indicate that the association between vaccination and increased health care use is not due to adverse events related to the vaccination. As the same vaccines were used during the entire study period, there is no obvious biological explanation for this time dependent change. Intense media attention concerning the potential adverse effects of the HPV vaccine has been seen in Denmark since 2013. This massive media attention could potentially have led to an increased awareness of potential symptoms, but it could also have made the girls and their parents more inclined to draw a link between experienced symptoms and the HPV vaccine. This might have increased their consultation rate, which could partly explain the results.

A recently published study by Héquet at al.[23] found that the use of medical services was a strong driver for HPV vaccination initiation at the individual level. This finding is in line with our results of the difference in health care utilization pattern between vaccinated and non-vaccinated girls prior to vaccination. We are not aware of other studies investigating the association between the HPV vaccine and later primary health care utilization. However, our findings are compatible with the findings reported in other post-licensure epidemiological studies, where no safety concerns have been detected [9–11,24,25].

One important strength of the study is the prospective study design and the large study population. The study population consisted of almost all girls born in Denmark in the period from 1996 to the end of 2002, with practically no loss to follow-up. Furthermore, all information at the individual level was obtained from national registers, which eliminates the risk of recall bias.

A limitation of the study is the conditioning on the future. Hence, in the study we only include girls born in Denmark, who were alive and living in Denmark at their 14^th^ birthday or the end of follow up. However, less than 0.5% (918) of girls died or emigrated between their 11^th^ and 14^th^ birthday and as the risk of both emigration and death are thought to be independent of HPV vaccination and health care attendance, the risk of bias is considered to be very limited. Another limitation in the study is the possibility of missing registrations on the given vaccinations. Still, as the reimbursement of the GPs depends on the registrations in the NHSR, severe underreporting is less likely. A recently published study, however, found that MMR vaccination overage at fifteen months of age in Denmark was higher than reported in the NHSR [26]. These potential administration errors could cause some misclassification of vaccination status in the current study. This is mainly a concern related to the MMR vaccine as the HPV vaccine was given three times, and it is considered less likely that the registration goes wrong all three times. Until 2014, the HPV only vaccinated girls had significantly higher consultation rates in the nine months before vaccination compared to the HPV+MMR and MMR only vaccinated girls. This might partly be explained by missing registrations of the MMR vaccines given. Hence, in the group of girls who had received both the HPV and MMR vaccine 31% of the girls had the vaccinations at different occasions and in 85% of the cases the MMR vaccine was given earlier then the HPV vaccine. Therefore, a missing registration of MMR vaccination (with a potential registration of a consultation instead) is thought to occur mainly before HPV only vaccination. The higher consultation rate for the HPV only vaccinated girls before vaccination was not present in 2014/2015. This might be due to the intense public debate about the HPV vaccine in 2013, which led to more strict registration of the vaccinations given in general practice.

In this study, a service code for a consultation recorded in the NHSR was disregarded in the analyses if it appeared in the same week as a vaccination was given. This was done because it was considered more likely to be an administrative error than an indication of morbidity. As the service codes are only recorded weekly in the NHRS, it was not possible to specifically disregard only the consultation codes on the specific day of vaccination. A consultation service code was registered along with a vaccination service code in approximately 15% of the vaccination weeks. Some of these registered consultations are likely not to be due to administrative errors, but to reflect actual consultations with the GP. Thus, there is a risk of underestimation of the health care utilization among the vaccinated girls in the same week as the vaccination took place.

Unmeasured confounding may be a limitation in the present study. Non-vaccinated girls may be different from vaccinated girls with respect to other characteristics. As an example, comorbidity may be linked to both vaccination status and consultation rates. However, due to the lack of information on comorbidity, we could not adjust for comorbidity in this study. Also, as a consequence of the media attention concerning possible adverse effects of the HPV vaccine starting in 2013, the HPV vaccination coverage decreased sharply in 2014. Hence, the distribution of covariates in the three vaccination groups, particularly in the non-vaccinated group, has probably changed. This potential variation in the distribution of unmeasured confounders could partly explain some of the differences in the results observed between those vaccinated before and those vaccinated after January 2014.

In our study, GP attendance was used as indicator for morbidity of the included girls. Unfortunately, the specific reasons for GP contact or potential diagnosis are not stated in the NHSR. Therefore, GP attendance is a crude measure of morbidity, and more severe morbidity might not be captured in our study as health care use in secondary care may not necessarily be associated with more GP contacts.

## Conclusion

In this study, no exclusive increase in health care utilization was detected as an indicator for morbidity after HPV vaccination. However, a general difference in the health care utilization pattern was found between vaccinated and non-vaccinated 12-year-old girls in the Danish childhood immunization program. A difference in health care utilization pattern between vaccinated and non-vaccinated girls was present already before vaccination, but increased after the time of vaccination, primarily for girls vaccinated in 2014/2015. This might reflect a general difference in the health care utilization between vaccinated and non-vaccinated girls and/or increased awareness of potential adverse effects after intense media attention from 2014 onwards.

To our knowledge, this study is the first of its kind. Hence, our results need to be further explored.
